# Synthesis and Supercapacitor Performance of Polyaniline/Nitrogen-Doped Ordered Mesoporous Carbon Composites

**DOI:** 10.1186/s11671-018-2577-3

**Published:** 2018-05-24

**Authors:** Kangjun Xie, Manman Zhang, Yang Yang, Long Zhao, Wei Qi

**Affiliations:** 1 School of Chemistry and Chemical Engineering, Huazhong University of Science and Technology, Wuhan, 430074 China; 2 Institute of Applied Electromagnetic Engineering, Huazhong University of Science and Technology, Wuhan, 430074 China

**Keywords:** Supercapacitor, Polyaniline, Ordered Mesoporous Carbon, Specific Capacitance

## Abstract

The electrochemical property of ordered mesoporous carbon (OMC) can be changed significantly due to the incorporating of electron-donating heteroatoms into OMC. Here, we demonstrate the successful fabrication of nitrogen-doped ordered mesoporous carbon (NOMC) materials to be used as carbon substrates for loading polyaniline (PANI) by in situ polymerization. Compared with NOMC, the PANI/NOMC prepared with a different mass ratio of PANI and NOMC exhibits remarkably higher electrochemical specific capacitance. In a typical three-electrode configuration, the hybrid has a specific capacitance about 276.1 F/g at 0.2 A/g with a specific energy density about 38.4 Wh/kg. What is more, the energy density decreases very slowly with power density increasing, which is a different phenomenon from other reports. PANI/NOMC materials exhibit good rate performance and long cycle stability in alkaline electrolyte (~ 80% after 5000 cycles). The fabrication of PANI/NOMC with enhanced electrochemical properties provides a feasible route for promoting its applications in supercapacitors.

**Electronic supplementary material:**

The online version of this article (10.1186/s11671-018-2577-3) contains supplementary material, which is available to authorized users.

## Background

With the aggravation of environmental pollution and resource shortage, the development and application of novel clean energy and energy storage become an urgent problem to be solved. As a new type of energy storage, the supercapacitor has attracted wide attention because of its fast charge and discharge rate, high power density, long cycle life, and non-pollution [1–3]. However, compared with traditional energy storage devices such as lithium-ion batteries, the low energy density of supercapacitor makes its application subject to many limitations [4–6]. The electrode materials are the most important factor affecting the performance of supercapacitor. Therefore, the research for a new high-performance electrode material has become a hotspot in the field of supercapacitor.

Polyaniline (PANI) is a typical conductive polymer material with low cost, easy synthesis, good conductivity, and high theoretical specific capacitance [7–10]. However, the performance of the PANI electrode will be significantly worse in the charge and discharge process, which is due to swelling and contraction of PANI in this process. Therefore, combining with electric steadily carbonaceous materials has become a wise method to improve the specific capacitance and cycling stability of PANI electrode. For example, Hao et al. [11] reported that boron-doped graphene was used as a high surface support for PANI deposition. A sandwich-like PANI/boron-doped graphene was obtained, which exhibits high specific capacitances and good electrochemical lifetime in both acidic and alkaline electrolytes during long-term cycling. Zhang et al. [12] reported that doping ordered mesoporous carbon with electron-donating nitrogen and sulfur heteroatoms to enhance its electrochemical performance.

Among the carbonaceous materials, mesoporous carbon material as a typical carbon material is widely used in adsorption, catalysis, electrochemistry, and other fields because of good surface area, adjustable ordered pore structure, uniform pore size, good chemical stability, high mechanical strength, and good conductivity [13–17]. In this article, we used nitrogen-doped ordered mesoporous carbon (NOMC) as framework for loading PANI by in situ polymerization to synthesize PANI/NOMC composites. Compared with individual components, the PANI/NOMC exhibits remarkably changed electrochemical specific capacitance. The specific capacitance of the hybrid can reach 276.1 F/g in 6 M KOH at 0.2 A/g in the three-electrode system. Meanwhile, the hybrid delivers an energy density about 38.4 Wh/kg at the power density around 200 W/kg. Moreover, PANI/NOMC materials exhibit good rate performance and long cycle stability in alkaline electrolyte (~ 80% after 5000 cycles).

## Materials and Methods

### Materials Synthesis

All the chemicals were analytical grade and used as received without further purification. Resol was synthesized from phenol and formaldehyde by stepwise polymerization as the following process [18]: first, phenol (0.94 g) was melted at 42 °C; next, 0.2 g of NaOH solution (20 wt%) was added slowly with stirring; then, 1.62 g of formaldehyde solution (37 wt%) was added dropwise and stirred for 1 h at 70 °C; and after cooling to room temperature, pH value was adjusted to 7.0 with 0.1 M HCl. Finally, the resol was obtained after vacuum drying at 50 °C.

For the typical synthesis of the NOMC [19], SBA-15 (0.33 g) was first dissolved in ethanol (9 g), 3 g of resol ethanol solution (20 wt%) was added, and then nitrile ammonia (0.3 g) were added and stirred for 8 h. Yellow powders were obtained by pouring the solution into a beaker to evaporate the solvent at 60 °C for 10 h. Next, yellow powders were added to a tubular furnace under N_2_ atmosphere at 800 °C for 3 h with a ramp rate of 10 °C/min. After cooling down to the room temperature, powders were dissolved in hydrofluoric acid (10 wt%). Then, the sample was filtered and washed with ethanol for several times. The final product was obtained after being dried in vacuum at 60 °C for 12 h.

In the synthesis of PANI/NOMC-*x* (*x* represents the initial mass ratio of PANI and NOMC), 0.1 g of NOMC was added into the mixture of ethanol (7.5 mL) and DMF (2.5 mL) for ultrasonic dispersion of a stable NOMC/ethanol/DMF suspension. Then, 0.1 xg aniline was dissolved in the NOMC/ethanol/DMF suspension under ice water bath with stirring for 2 h. Next, ammonium persulfate and hydrochloric acid (mole ratio of aniline/ammonium persulfate/HCl was 1:1:1) were added in suspension at ice water bath with stirring for 10 h. Then, the suspension was centrifuged at 8000 rpm for 20 min, discarding supernatant solution; the sediment was collected and washed with ethanol and deionized water several times. Finally, PANI/NOMC-*x* was obtained after being dried in vacuum at 50 °C for 1 h.

### Materials Characterization

The morphology features of NOMC and PANI/NOMC-*x* were characterized by transmission electron microscopy (Tecnai G2 F30) and scan electron microscopy (Sirion 200). FT-IR spectra and X-ray powder diffraction were provided to the structure of NOMC and PANI/NOMC-*x*. X-ray photoelectron spectroscopy (XPS) was used to measure the mass ratio of C, N, and O in PANI/NOMC-*x*. The pore size and density of NOMC and PANI/NOMC-*x* were measured through a Brunauer–Emmett–Teller (BET) experiment at N_2_ condition.

### Electrochemical Measurement

The electrochemical properties of the materials were performed with an electrochemical analyzer-CHI 660E (Shanghai, Chenhua Limited Co.) under ambient conditions in KOH (2 M) aqueous solution, using a three-electrode system with PANI/NOMC-*x* as the working electrode, a platinum wire as the counter electrode, and a saturated calomel electrode as the reference electrode. The working electrode was prepared by mixing the PANI/NOMC-*x*, acetylene black, and polytetrafluoroethylene with the mass ratio 85:10:5. The mixture was coated onto current collectors (1.0 cm^2^), pressed at 10 MPa, and dried under vacuum at 50 °C. According to some reports [20, 21], the specific capacitance can be calculated from galvanostatic charge/discharge curves by Eq. (1) and the power density and the energy density calculated by Eqs. (2) and (3), respectively

1 $$ C= It/\left(\varDelta Vm\right) $$

2 $$ E=1/2 C\varDelta {V}^2 $$

3 $$ P=E/t $$

## Results and Discussion

**Fig. 1:**
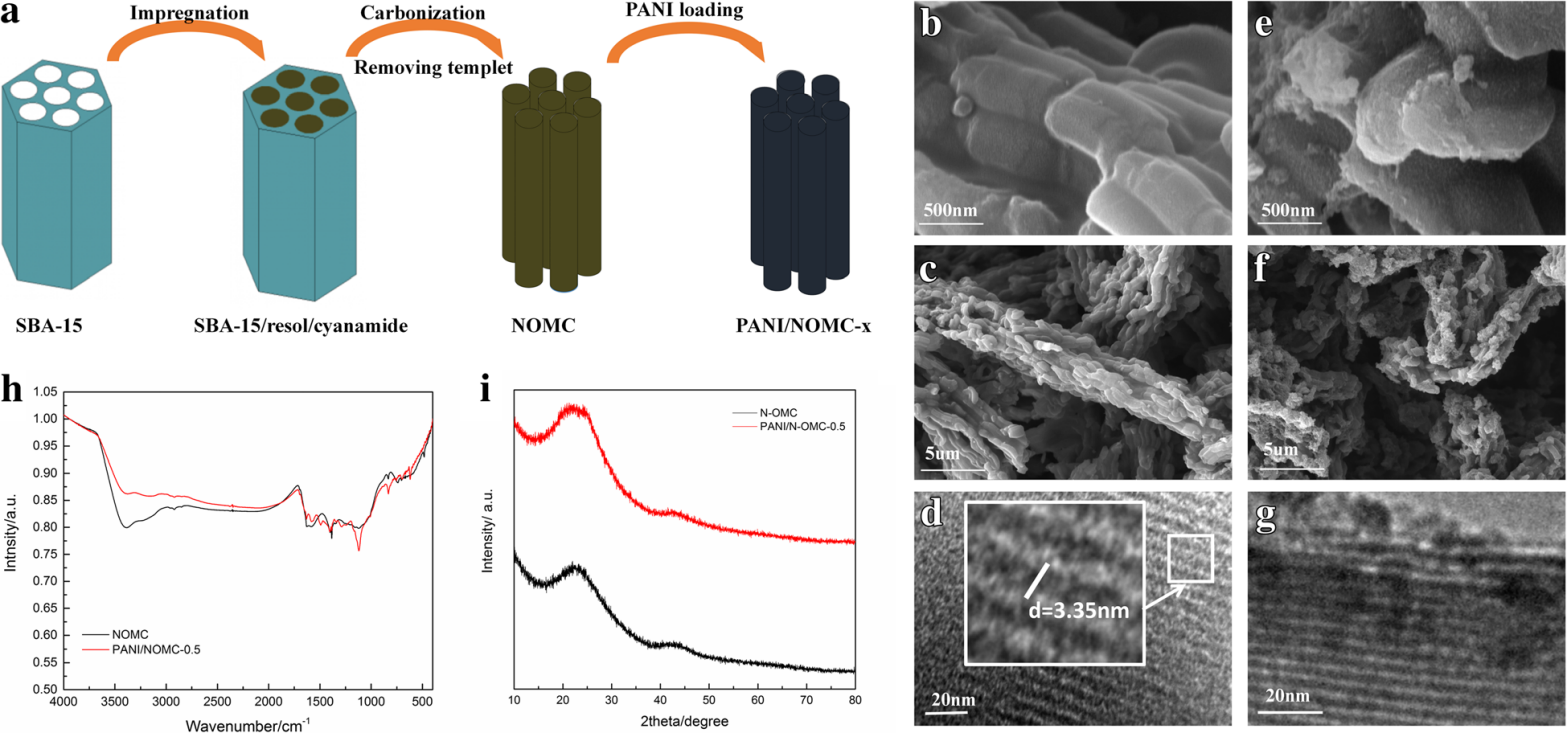
Scheme for the fabrication of PANI/NOMC-*x* (**a**). SEM images of NOMC (**b**, **c**) and PANI/NOMC-0.5 (**e**, **f**). TEM images of NOMC (**d**) and PANI/NOMC-0.5 (**g**). FT-IR spectra (**h**) and XRD patterns (**i**) of NOMC and PANI/NOMC-0.5

The synthesis process of PANI/NOMC-*x* is shown in Fig. 1a. Resol and cyanamide were injected into SBA-15, and then, the hybrids were carbonized at 800 °C, and next, the hybrids were added into the HF aqueous solution (10 wt%) to remove the templet to obtain the PANI/NOMC-*x*. The morphologies of NOMC and PANI/NOMC-*x* are also shown in Fig. 1. SEM images of a typical sample of NOMC (Fig. 1b, c) and PANI/NOMC-0.5 (Fig. 1e, f) reveal that NOMC and PANI/NOMC-0.5 consist of many cylindrical particles with uniform sizes of 1 μm. The coating layers on the surface of PANI/NOMC-0.5 indicate the successful coating of PANI on the surface of NOMC. The TEM image of NOMC (Fig. 1d) clearly displays uniform stripe-like arranged images, and the stripe spacing is about 3 nm. After coating with PANI, we can also see the uniform stripe-like arranged images in the TEM image of PANI/NOMC-0.5 (Fig. 1g and Additional file 1: Figure S3), indicating that coating with PANI would not change the pore structure of NOMC.

**Fig. 2:**
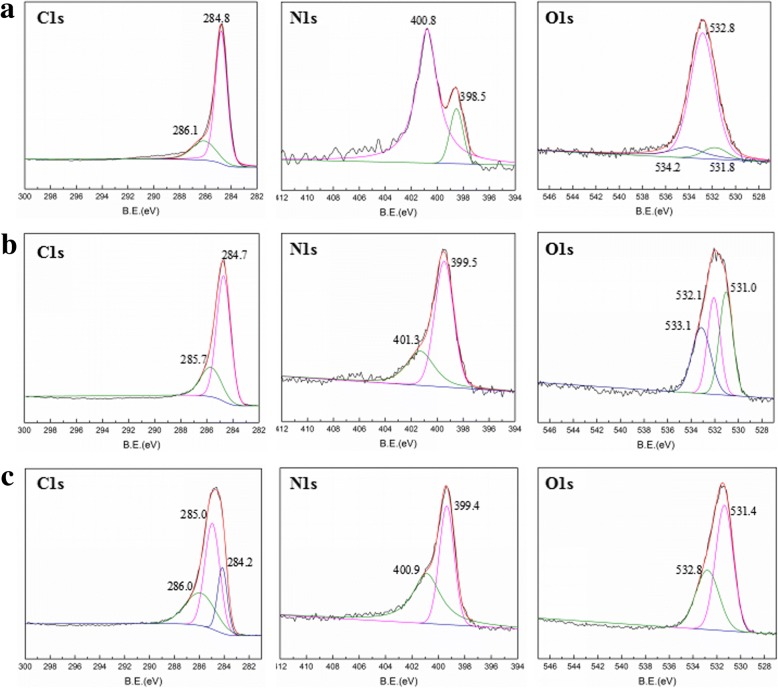
XPS spectra of C_1s_, N_1s_, and O_1s_ for NOMC (**a**), PANI/NOMC-0.5(**b**), and PANI/NOMC-4 (**c**)

**Fig. 3:**
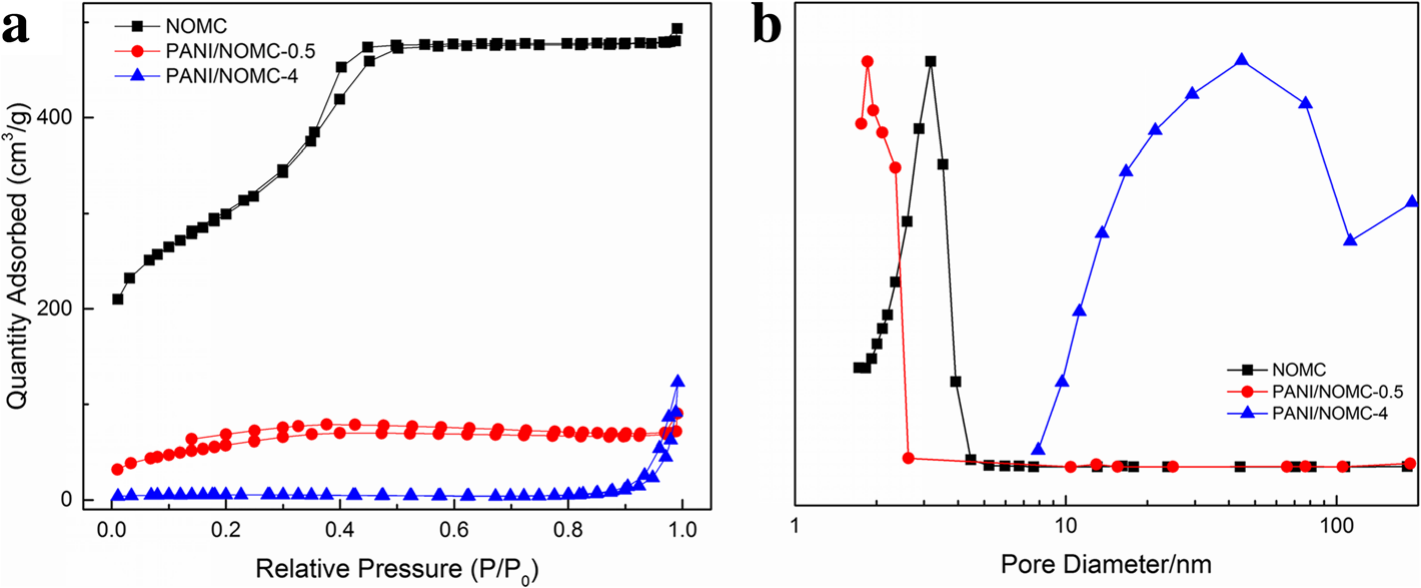
N_2_ adsorption–desorption isotherms of NOMC, PANI/NOMC-0.5, and PANI/NOMC-4 (**a**). Pore size distribution of NOMC, PANI/NOMC-0.5, and PANI/NOMC-4 (**b**)

The FT-IR spectra of NOMC and PANI/NOMC-*x* are shown in Fig. 1h and Additional file 1: Figure S1. It can see the characteristic adsorption peak of PANI at 1120 cm^−1^ and that of PANI/NOMC-*x* at 1300 and 1496 cm^−1^, respectively. These peaks can be attributed to the stretching vibration of N=Q=N, C–H, and C=C of benzenoid units. As the mass ratio of PANI increases, the intensity of these peaks is strongly increasing (Additional file 1: Figure S1), which indicates further that PANI was coated on NOMC successfully. From the XRD patterns of NOMC and PANI/NOMC-0.5 (Fig. 1i), we can see NOMC and PANI/NOMC-0.5 are atypical carbon, suggesting the coating of PANI would not change the structure of NOMC. The XPS results showed the atomic environments and contents of C, N, and O in NOMC and PANI/NOMC-*x* (Fig. 2 and Table 1). As well known, the oxygen/nitrogen functionalities based on O_1s_ spectra (524–540 eV) and N_1s_ spectra (about 400 eV) are very single, through which we can calculate the O and N content of the composites but not reflect the combining way of C, O, and N. Thus, the C_1s_ spectra are analyzed to reflect the environment of C, N, and O atoms. For the C_1s_ spectra of NOMC, the C_1_ (248.8 eV) might be attributed to the π-π* transition in C=C sp^2^ delocalized bonds, and C_2_ reflects the bonds of C=O from carbonyl or carboxylic [22]. As previous reports, the N elements are fitted into five species: pyridinic nitrogen species at 398.4 eV, amino nitrogen species at 399.3 eV, pyrrolic nitrogen species at 400.2 eV, and the species at 401.1 and 403.5 eV assigned to graphitic and N^+^–O^−^ nitrogen, respectively [23]. Almost all N_1s_ species of NOMC at 400.8 eV were very close to the graphitic nitrogen species of 401.1 eV (Fig. 2 and Table 1). Therefore, the synthesis mechanism of NOMC can be speculated as the following: the thermal decomposition of C and N atoms from resol and nitrile ammonia can be carbonized to NOMC through the template of SAB-15 at high temperature (800 °C) with the forming of the high stable bonds of graphitic nitrogen (C–N) [24, 25]; meanwhile, the formation of C=O may be attributed to the existence of O atoms of resol; anyway, compared with the single OMCs, the N-doped OMCs will have a large surface area with high mesoporosity and so as to a specific capacitance and good rate capability [19]. In addition, with the mass ratio of PANI in PANI/NOMC-*x* increasing, the content of C_1_ decreased from 62.60 to 39.83% and that of C_2_ increased gradually (Table 1), which indicates that the bonds of C=C broke during the producing of the composites, informing the PANI/NOMC-*x* is synthesized successfully further. What is more, according to the N content of PANI/NOMC-*x* increases, there is more PANI coated on the surface of NOMC with the mass ratio increasing. Interestingly, when the mass ratio of PANI increased up to 0.5 to 4, the O content of PANI/NOMC-*x* increased suddenly; it might be reasoned that the excess PANI reacted with persulfate during the production of composites, and then, the reacted produce was coated on the surface of NOMC; the enhancive O content for PANI/NOMC-x may impact on their electrochemical performance. In addition, the BET of NOMC and PANI/NOMC-*x* were carried out through nitrogen adsorption–desorption isotherm experiments under a temperature of − 200 °C (Fig. 3 and Additional file 1: Figure S3); the BET surface area of NOMC, PANI/NOMC-0.2, PANI/NOMC-0.5, PANI/NOMC-1, PANI/NOMC-2, and PANI-NOMC-4 are 1051.31, 530.20, 209.39, 178.10, 26.15, and 18.05 m^2^/g, respectively, and the adsorption average pore size of those are 2.82, 3.00, 2.12, 2.61, 10.23, and 31.30 nm, respectively. The decreasing BET surface area for the composites can be the result of the coating of PANI on the surface of NOMC. The larger pore size for PANI/NOMC-4 than that for PANI and PANI/NOMC-0.5 can be explained that the coating PANI blocks the pores of NOMC, and the blockage effect is more serious with the content of PANI increasing till the pores of NOMC are blocked completely; therefore, the increased pore size of PANI/NOMC-4 may be the space between the coated PANI, and this result agreed with the capacitance changes of PANI/NOMC-*x* in the following investigation.

**Table 1:** The XPS characterization of NOMC, PANI/NOMC-0.5, and PANI/NOMC-4

Sample ID		C_1s_			N_1s_		O_1s_		
NOMC	B.E. (eV)	284.8	286.1	–	398.5	400.8	531.8	532.8	534.2
	at.%	62.60	19.01	0	0.70	4.06	0.99	11.10	1.53
PANI/NOMC-0.5	B.E. (eV)	284.7	285.7	–	399.5	401.3	531.0	532.1	533.1
	at.%	52.46	18.33	0	8.48	4.18	5.97	5.26	5.33
PANI/NOMC-4	B.E. (eV)	284.2	285.0	286.0	399.4	400.9	531.4	532.8	–
	at.%	13.49	26.34	17.22	6.47	7.37	17.99	11.12	0

**Fig. 4:**
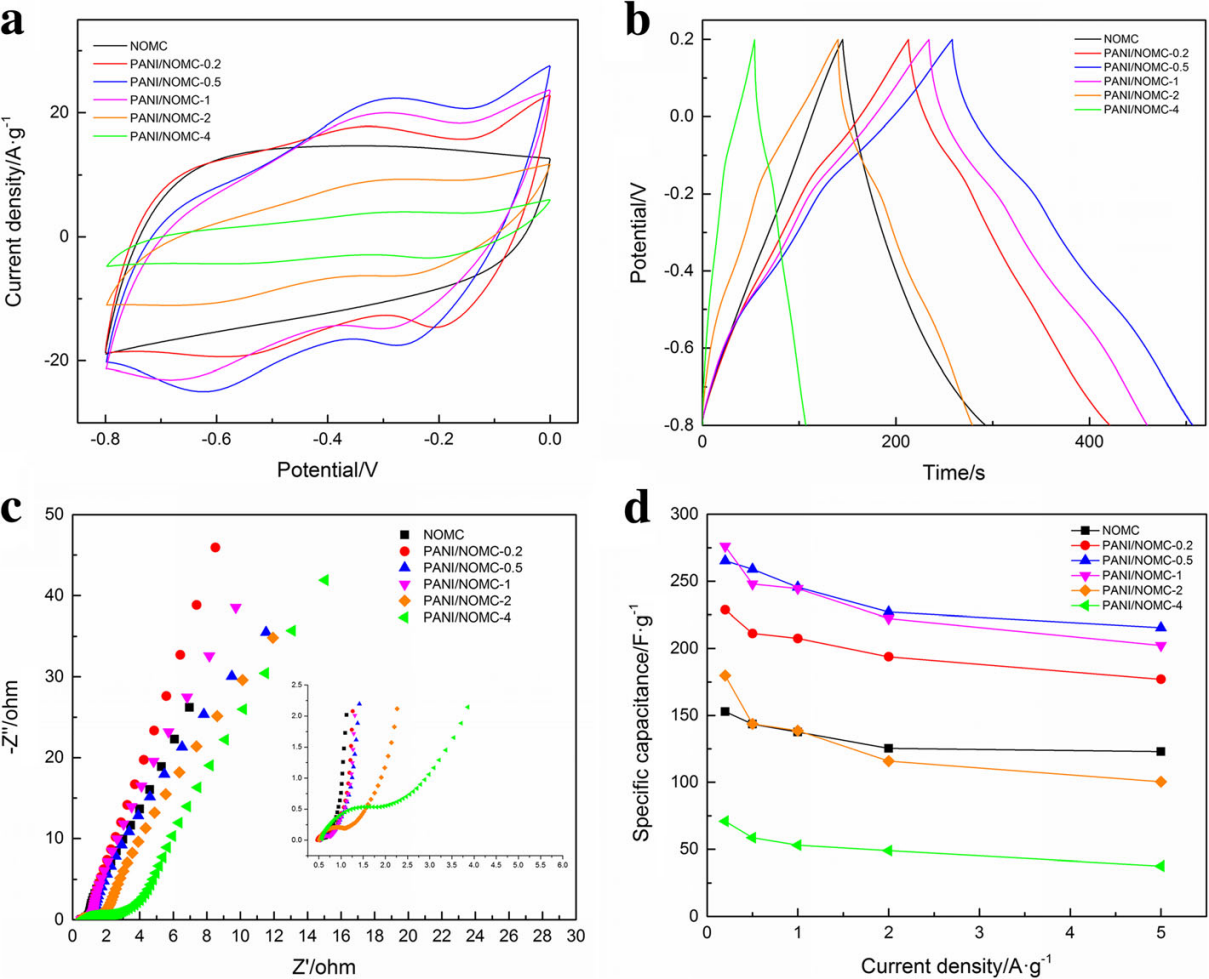
CV curves of NOMC and PANI/NOMC-*x* at a scan rate of 0.1 V/s (**a**) Galvanostatic charge/discharge curves of NOMC and PANI/NOMC-*x* at a current density of 1 A/g (**b**). Nyquist plots of NOMC and PANI/NOMC-*x* (**c**). Specific capacitance of NOMC and PANI/NOMC-*x* electrodes with different current densities (**d**). 0.6 M KOH was used as the electrolyte for all tests

The electrochemical performance of NOMC and PANI/NOMC-*x* was evaluated using a cyclic voltammetry (CV) method. As shown in Fig. 4a, NOMC and PANI/NOMC-*x* present an approximately rectangular CV shape at a scan rate of 0.1 V/s, which is the typical feature of a double-layer capacitor. For PANI/NOMC-*x*, the CV curve exhibits two pairs of redox peaks because of the redox transition of PANI between leucoemeraldine/emeraldine/pernigraniline structural conversions [11]. Figure 4b shows the galvanostatic charge-discharge curves of NOMC and PANI/NOMC-*x* electrodes measured at a current density of 1 A/g. The specific capacitance of NOMC, PANI/NOMC-0.2, PANI/NOMC-0.5, PANI/NOMC-1, PANI/NOMC-2, and PANI/NOMC-4 calculated from the discharge curves are 137.6, 211.2, 258.9, 244.5, 143.6, and 53.0 F/g, respectively. With the increase of the mass ratio of PANI, the specific capacitance of PANI/NOMC-*x* was first rising and then dropping. It can be due to that less PANI will provide faradaic pseudo-capacitance to increase the specific capacitance of PANI/NOMC-*x*, but with more PANI coated onto NOMC, the pore structure will be blocked so as to decrease the BET surface of composites and then leading to the lower specific capacitance gradually. Figure 4c shows the Nyquist plot of NOMC and PANI/NOMC-*x*. All of the PANI/NOMC-*x* materials show a small semicircle in the high-frequency region, which is caused by the charge transfer resistance at the interface between the electrode and electrolyte, indicating that PANI/NOMC-*x* composites have good electrical conductivity. In the low-frequency region, the slope of all these curves is very large; it may indicate PANI/NOMC-*x* have great capacitive performance according to the report [22]. Figure 4d shows the specific capacitance of NOMC and PANI/NOMC-*x* in different current densities. With the increase of current density, the specific capacitance of NOMC and PANI/NOMC-*x* decreases slowly. When current density increased 25 times from 0.2 to 5 A/g, the specific capacitance of PANI/NOMC-0.5 is decreased only from 265.3 to 215.5 F/g (about 81.2% retained), demonstrating PANI/NOMC-0.5 has good rate performance.

**Fig. 5:**
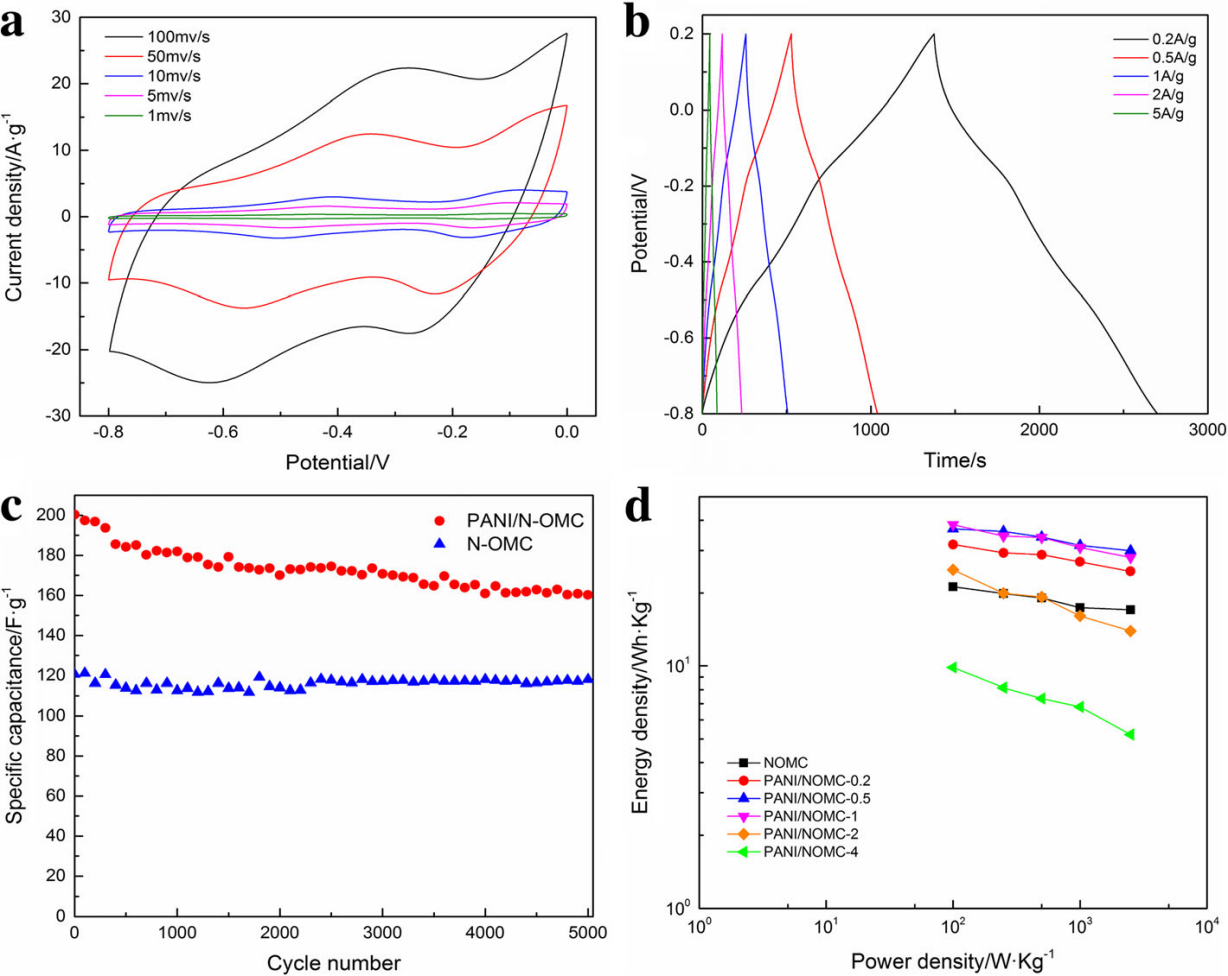
CV curves of PANI/NOMC-0.5 (**a**). Galvanostatic charge/discharge curves of PANI/NOMC-0.2 (**b**). The cycling performance of PANI/NOMC-0.5 in 6 M KOH at 5 A/g about 5000 cycles (**c**). The Ragone plots of NOMC and PANI/NOMC-*x* (**d**)

CV curves of NOMC and PANI/NOMC-*x* at different scan rates are shown in Fig. 5a and Additional file 1: Figure S2 a, c, e, and g. It can see the CV curve of NOMC is approximately rectangular shape at all scan rates, indicating the capacitance of NOMC is double electrode layer capacitance. After coating with PANI, there are redox peaks in the CV curves of PANI/NOMC-*x* demonstrating that the capacitance of PANI/NOMC-*x* is determined by double electrode layer capacitance and faradaic pseudo-capacitance. Figure 5b and Additional file 1: Figure S2 b, d, f and h show the galvanostatic charge/discharge curves of NOMC and PANI/NOMC-*x*. It can be observed that PANI/NOMC-0.5 has the biggest specific capacitance compared with other materials. The cycling performance of NOMC and PANI/NOMC-0.5 is shown in Fig. 5c. It is easy to see that NOMC have excellent cycling performance for the capacitance retaining about 95% after 5000 cycles, which is better than that of PANI/NOMC-*x* composites. Interestingly, PANI/NOMC has a larger specific capacitance than that of NOMC in all the cyclic processes. The Ragone plots of NOMC and PANI/NOMC are shown in Fig. 5d, and the results are as follows: the energy density of PANI/NOMC-0.5 decreased hardly as the power density increased that is unusual phenomenon for other reports [20, 21], and the detailed mechanism should be investigated furtherly in the future. Anyway, the results of this work are of great significance to realize the application of supercapacitors in the industry.

## Conclusion

The PANI/NOMC composites were successfully synthesized by hard template with in situ polymerization. By combining the PANI with high theoretical specific capacitance and the NOMC with good cycle stability, it solves the problem that the capacitance of electric double-layer capacitor is small and the cycle performance of pseudo-capacitance material is poor. PANI/NOMC composites exhibit big specific capacitance, good rate performance, and long cycle stability with excellent application prospects. Through this work, it might provide some basic data for promoting the application of flexible supercapacitors in wearable equipment.

## Additional file

Additional file 1: **Figure S1.** FT-IR spectra of PANI/NOMC-*x* materials. **Figure S2.** CV curves of PANI/NOMC-0.2 (a), PANI/NOMC-1 (c), PANI/NOMC-2 (e), and PANI/NOMC-4 (g) at different scan rates; galvanostatic charge/discharge curves of PANI/NOMC-0.2 (b), PANI/NOMC-1 (d), PANI/NOMC-2 (f), and PANI/NOMC-4 (h) at different current densities. **Figure S3.** N_2_ adsorption–desorption isotherms of NOMC, PANI/NOMC-0.2, PANI/NOMC-0.5, PANI/NOMC-1, PANI/NOMC-2, and PANI/NOMC-4 (a); pore size distribution of PANI/NOMC-0.2, PANI/NOMC-1, and PANI/NOMC-2. (DOCX 3025 kb)

## Abbreviations

DMF: Dimethylformamide
NOMC: Nitrogen-doped ordered mesoporous carbon
OMC: Ordered mesoporous carbon
PANI: Polyaniline
PANI/NOMC-*x*: Composites of nitrogen-doped ordered mesoporous carbon and polyaniline with different mass ratios
SEM: Scanning electron microscope
TEM: Transmission electron microscopy
XPS: X-ray photoelectron spectroscopy
XRD: X-ray powder diffraction
