# Supplementary material for: Synthesis and Supercapacitor Performance of Polyaniline/Nitrogen-Doped Ordered Mesoporous Carbon Composites
Source: Nanoscale Res Lett. 2018 May 24;13:163. doi: 10.1186/s11671-018-2577-3 (PMC5968012; doi:10.1186/s11671-018-2577-3)
Supplement: Supplementary file 1 — Figure S1. FT-IR spectra of PANI/NOMC-x materials. Figure S2. CV curves of PANI/NOMC-0.2 (a), PANI/NOMC-1 (c), PANI/NOMC-2 (e), and PANI/NOMC-4 (g) at different scan rates; galvanostatic charge/discharge curves of PANI/NOMC-0.2 (b), PANI/NOMC-1 (d), PANI/NOMC-2 (f), and PANI/NOMC-4 (h) at different current densities. Figure S3. N2 adsorption–desorption isotherms of NOMC, PANI/NOMC-0.2, PANI/NOMC-0.5, PANI/NOMC-1, PANI/NOMC-2, and PANI/NOMC-4 (a); pore size distribution of PANI/NOMC-0.2, PANI/NOMC-1, and PANI/NOMC-2. (DOCX 3025 kb) [file 11671_2018_2577_MOESM1_ESM.docx]

**Supplementary Information**

**Synthesis and electrochemical applications of** **polyaniline/nitrogen-doped ordered** **mesoporous carbon composites**

Kangjun Xie^1,2^, Manman Zhang^1,2^, Yang Yang^1,2^, Long Zhao^2^, Wei Qi^1,2*^

^a^School of Chemistry and Chemical Engineering, Huazhong Universityof Science and Technology, Wuhan 430074, China

^b^Institute of Applied Electromagnetic Engineering, Huazhong University of Science and Technology, Wuhan 430074, China

^*^Corresponding author at: School of Chemistry and Chemical Engineering, Huazhong University of Science and Technology, Wuhan 430074, China(W.Qi).

Email addresses:

[xiekangjun@hust.edu.cn](mailto:xiekangjun@hust.edu.cn) (Kangjun Xie)

[2991969359@qq.com](mailto:2991969359@qq.com) (Manman Zhang)

[812396212@qq.com](mailto:812396212@qq.com) (Yang Yang)

[zhaolong@hust.edu.cn](mailto:zhaolong@hust.edu.cn) (Long Zhao)

[qiwei@hust.edu.cn](mailto:qiwei@hust.edu.cn) (Wei. Qi)


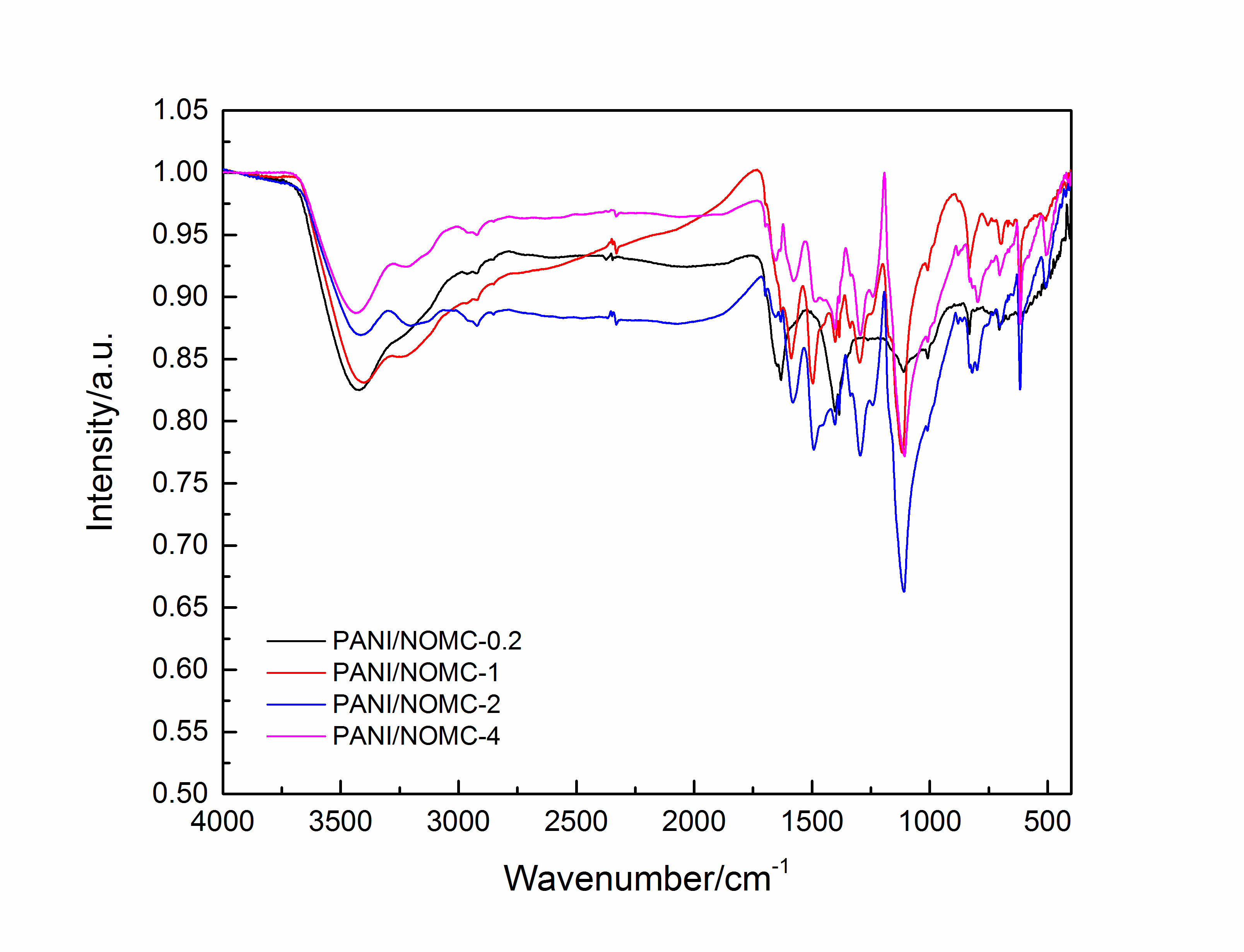


Figure S1 FT-IR spectra of PANI/NOMC-x materials.


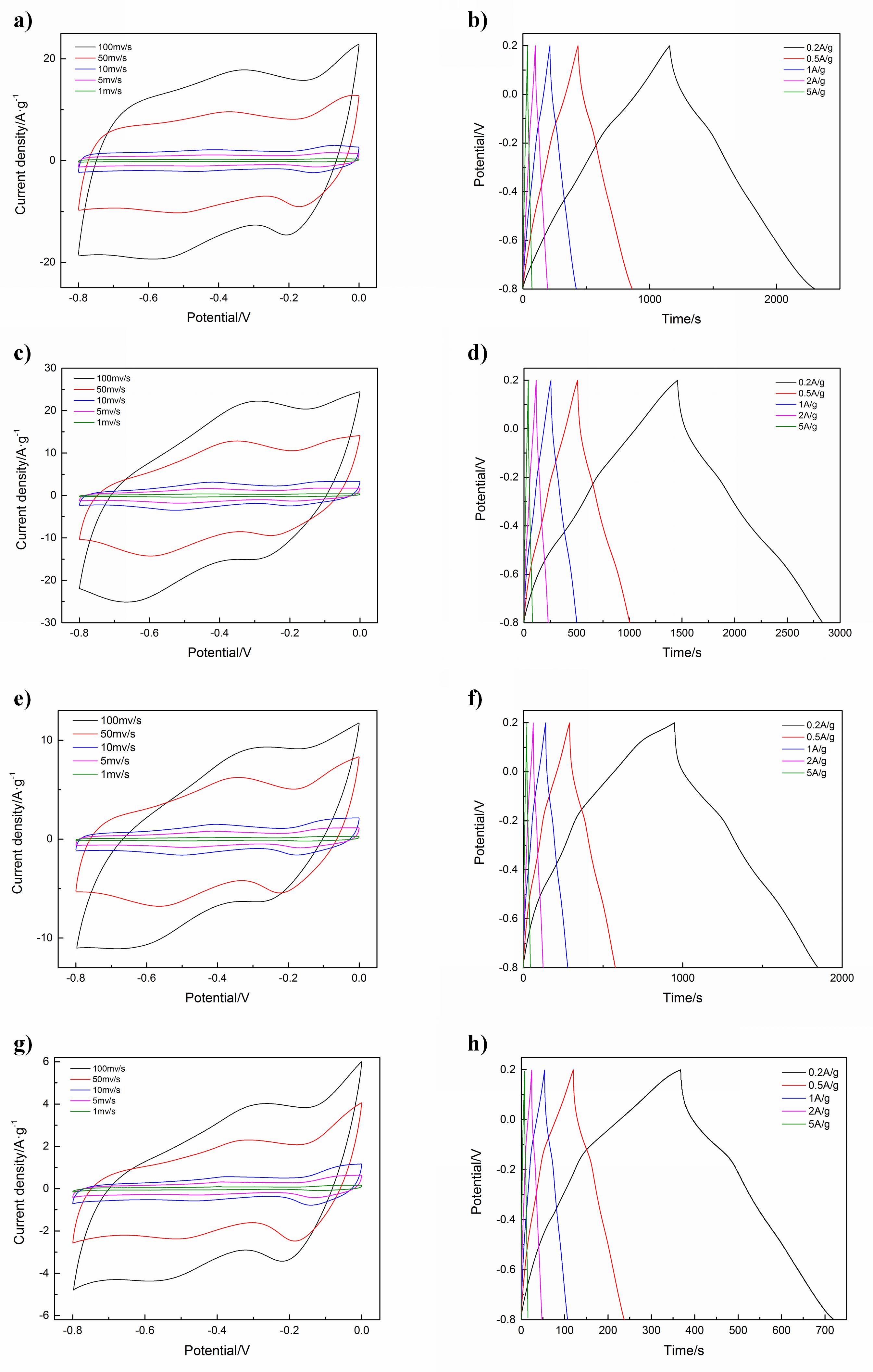


Figure S2 CV curves of PANI/NOMC-0.2 (a), PANI/NOMC-1 (c), PANI/NOMC-2 (e) and PANI/NOMC-4 (g) at different scan rate; Galvanostatic charge/discharge curves of PANI/NOMC-0.2 (b), PANI/NOMC-1 (d), PANI/NOMC-2 (f) and PANI/NOMC-4 (h) at different current density.


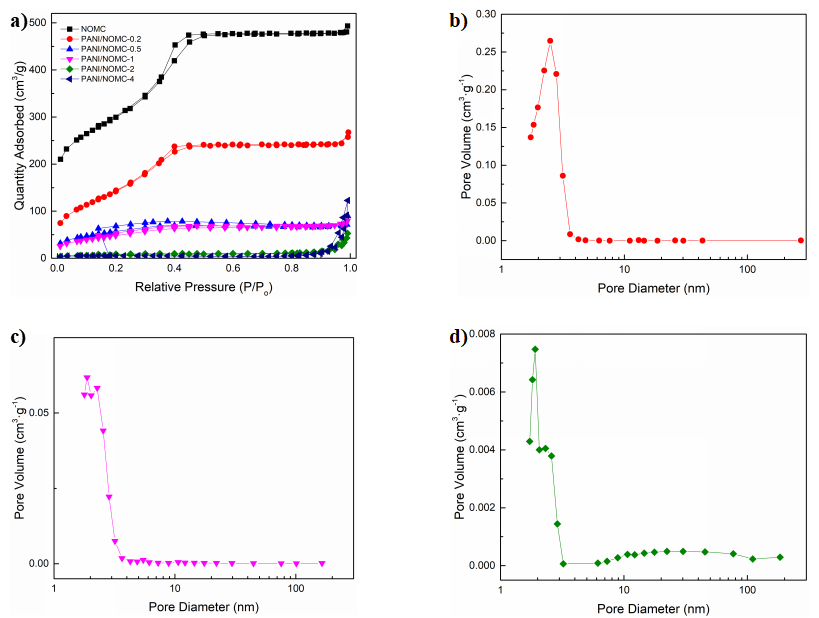


Figure S3 N_2_ adsorption–desorption isotherms of NOMC, PANI/NOMC-0.2, PANI/NOMC-0.5, PANI/NOMC-1, PANI/NOMC-2 and PANI/NOMC-4 (a); pore size distribution of PANI/NOMC-0.2, PANI/NOMC-1 and PANI/NOMC-2.
